# Using Self-Assembling Peptides to Integrate Biomolecules into Functional Supramolecular Biomaterials

**DOI:** 10.3390/molecules24081450

**Published:** 2019-04-12

**Authors:** Renjie Liu, Gregory A. Hudalla

**Affiliations:** J. Crayton Pruitt Family Department of Biomedical Engineering, Wertheim College of Engineering, University of Florida, Gainesville, FL 32611, USA; renjieliu@ufl.edu

**Keywords:** self-assembly, nanomaterials, peptides, carbohydrate, proteins

## Abstract

Throughout nature, self-assembly gives rise to functional supramolecular biomaterials that can perform complex tasks with extraordinary efficiency and specificity. Inspired by these examples, self-assembly is increasingly used to fabricate synthetic supramolecular biomaterials for diverse applications in biomedicine and biotechnology. Peptides are particularly attractive as building blocks for these materials because they are based on naturally derived amino acids that are biocompatible and biodegradable; they can be synthesized using scalable and cost-effective methods, and their sequence can be tailored to encode formation of diverse architectures. To endow synthetic supramolecular biomaterials with functional capabilities, it is now commonplace to conjugate self-assembling building blocks to molecules having a desired functional property, such as selective recognition of a cell surface receptor or soluble protein, antigenicity, or enzymatic activity. This review surveys recent advances in using self-assembling peptides as handles to incorporate biologically active molecules into supramolecular biomaterials. Particular emphasis is placed on examples of functional nanofibers, nanovesicles, and other nano-scale structures that are fabricated by linking self-assembling peptides to proteins and carbohydrates. Collectively, this review highlights the enormous potential of these approaches to create supramolecular biomaterials with sophisticated functional capabilities that can be finely tuned to meet the needs of downstream applications.

## 1. Introduction

**Figure 1:**
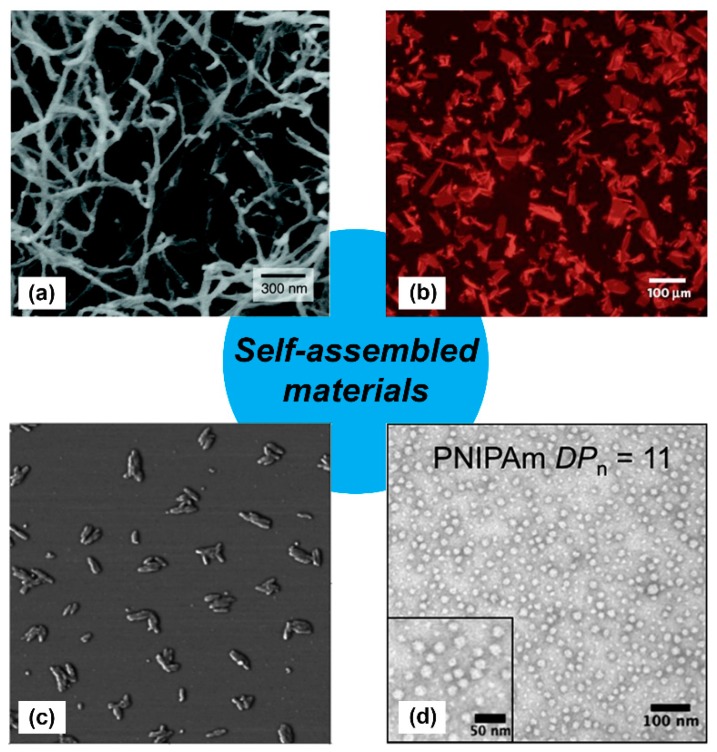
Examples of self-assembled materials with different morphologies. (**a**) Scanning electron micrograph of an isoluecine–lysine–valine–alanine–valine (IKVAV)-containing peptide nanofiber network formed by adding cell media (Dulbecco’s modified Eagle medium; DMEM) to an aqueous solution of a peptide amphiphile. Adapted with permission from [11]. Copyright 2004, Science publishing. (**b**) Fluorescence photomicrograph of individual two-dimensional (2D) crystalline sheets assembled from periodic amphiphilic peptoid polymers. Adapted with permission from [12]. Copyright 2010, Nature publishing. (**c**) Atomic force microscopy (AFM) phase image of small rods derived from self-assembling oligothiophenes prepared in *n*-butanol solution. Adapted with permission from [13]. Copyright 2004, American Chemical Society publishing. (**d**) Transmission electron microscopy image of self-assembled poly(*N*-isopropylacrylamide) nanoparticles in aqueous solution. Reproduced from reference [14] with permission from The Royal Society of Chemistry.

Organization of individual molecules into a higher-ordered supramolecular structure, normally referred to as “self-assembly” [1], is a hallmark of living systems that is increasingly being used to fabricate synthetic biomaterials [2,3,4,5,6,7]. Self-assembly is mediated by weak physical interactions between molecules, including hydrogen bonds, ionic bonds, hydrophobic interactions, and van der Waals interactions [8,9]. The accumulation of these weak interactions results in a stable ordered supramolecular structure [10], as witnessed through DNA hybridization and protein folding. This supramolecular order can establish unique functional properties. For example, folded proteins can catalyze reactions (e.g., enzymes) or recognize ligands (e.g., transmembrane receptors) with specificity and selectivity that are not seen when the same protein is denatured. Inspired by these natural examples, significant efforts are now focused on designing synthetic molecules, such as peptides, peptoids, oligomers, and polymers that can self-assemble into nano-scale structures with different morphologies (Figure 1) [11,12,13,14]. In turn, synthetic nanoparticles, nanofibers, and nano-vesicles fabricated via self-assembly can be employed as three-dimensional scaffolds, vehicles, and carriers for diverse applications, including drug delivery, tissue engineering, biosensors, stimuli-responsive materials, and vaccine development [12,15,16,17,18,19,20,21]. 

Synthetic peptides are central to the tremendous advances in self-assembled nanomaterials seen over the last few decades [6,22]. Peptides are particularly attractive as building blocks for biomedical nanomaterials because of the biocompatibility and biodegradability of amino acids. Furthermore, extensive research into the influence of amino-acid sequence on self-assembly has led to general guidelines for designing synthetic peptides that adopt predictable supramolecular structures [23,24,25]. Finally, peptides can be synthesized using scalable and cost-effective processes, such as solid-phase methods or ring-opening polymerization, or can be expressed from recombinant DNA using microbial systems [26,27]. With advances in synthetic methods, peptides with well-defined sequences can now be easily obtained in relatively high yields [28]. As a result, self-assembled nanomaterials based on α-helical peptides, β-sheet fibrillizing peptides, amphiphilic peptides, ionic-complementary peptides, long-chain alkylated peptides, and cyclic peptides have been reported to date [22,29,30,31,32,33,34]. Among them, β-sheet fibrillizing peptides can assemble into diverse nanostructures, including tubes, fibers, and spheres [35,36,37,38,39]. Building upon these advances in self-assembled material science, it is now possible to create peptide-based structures with functional properties that are useful for biomedical applications [40,41,42,43,44,45,46,47]. Central to this is the ability to incorporate biologically active molecules into the nanomaterial itself. This is often achieved by linking a ligand directly to a self-assembling building block [6,48]. This review highlights recent advances in the use of self-assembling peptides as tags to integrate functional biomolecules, such as peptides, carbohydrates, and proteins, into supramolecular biomaterials.

## 2. Peptide Assemblies as Scaffolds for Receptor-Binding Ligands

Interactions between cell surface receptors and their cognate ligands are important mediators of many biological processes. Short peptides excised from protein ligands or identified via high-throughput screens can often mimic the function of the native protein ligands. For example, the arginine–glycine–aspartic acid (RGD) peptide found in many extracellular matrix proteins binds to integrin receptors and can mediate cell adhesion when immobilized onto a biomaterial surface [49,50,51]. Inspired by this, peptides such as RGD [52], YHWYGYTPQNVI (GE11) [53], and VFDNFVLKK [54] are incorporated into peptide-based nanomaterials to encourage cell adhesion, enable targeted delivery, or stimulate cell differentiation into specific phenotypes through recognition and activation of specific cell surface receptors [11,55,56,57,58,59,60,61,62,63,64,65,66,67].

**Figure 2:**
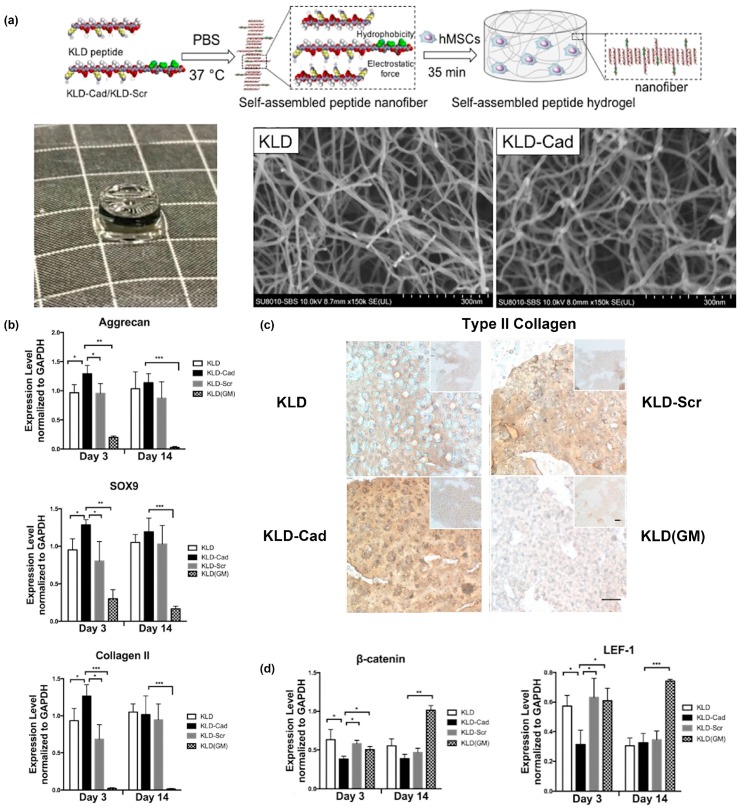
KLD-Cad hydrogels that promote chondrogenic differentiation of hMSCs. (**a**) Schematic depicting encapsulation of human mesenchymal stem cells (hMSCs) in self-assembled KLD- Cad/KLD-Scr hydrogels (top image). Picture of free-standing KLD-Cad self-assembled hydrogel (bottom left). Representative SEM images of fibrous structure within KLD (bottom middle) and KLD-Cad (bottom right) hydrogels. (**b**) Quantitative analysis of chondrogenic gene expression by hMSCs cultured in self-assembled peptide hydrogels after 3 and 14 days; hMSCs cultured on KLD-Cad-containing hydrogel showed significantly higher chondrogenic gene expression. (**c**) Histologic sections showing Type II collagen content in KLD, KLD-Cad, and KLD-Scr hydrogels after 14 days of chondrogenic culture compared to non-chondrogenic KLD hydrogels cultured in basal growth media. A notable higher amount of Type II collagen was observed in KLD-Cad-containing hydrogels compared with other groups. (**d**) Quantitative analysis of β-catenin and *LEF-1* gene expression by hMSCs. Gene expression was significantly inhibited by KLD-Cad hydrogels on day 3. *: *p* < 0.05, **: *p* < 0.01, ***: *p* < 0.001. Adapted with permission from [69]. Copyright 2017, Elsevier publishing.

One example from Bian and colleagues created a fusion of the *N*-cadherin mimetic peptide (HAVDI) and the self-assembling KLD peptide (Ac-KLDLKLDLKLDL), referred to as “KLD-Cad”, to fabricate self-assembled hydrogels that promote chondrogenesis of human mesenchymal stem cells (hMSCs) [68]. In a second paper, they elucidated the mechanism by which KLD-Cad hydrogels induced chondrogenesis of hMSCs [69]. Specifically, KLD-Cad peptide or KLD-Scr (Ac-AGVIDHGKLDLKLDLKLDL, used here as a negative control) was first mixed with KLD peptide in sterilized phosphate-buffered saline (PBS) to obtain precursor solutions. These mixtures were then incubated with hMSC precursor solutions and allowed to incubate at 37 °C for 35 min to form stable self-assembled hydrogels (Figure 2a). SEM images of the obtained free-standing hydrogels demonstrated no significant differences in the nanofibrous structure for KLD-Cad or KLD-Scr (Figure 2a). KLD-Cad-containing hydrogels induced higher chondrogenic gene expression levels (Figure 2b) and a greater amount of cartilaginous matrix (Figure 2c) after 14 days of in vitro culture of hMSCs. Lastly, canonical Wnt signaling was inhibited in cells cultured on KLD-Cad hydrogels but not KLD-Scr hydrogels, suggesting that knockdown of Wnt signaling promoted chondrogenic differentiation of hMSCs. (Figure 2d). Collectively, these studies highlight the promise of using functional peptide assemblies to control cell phenotype and function by engaging specific cell surface receptors.

**Figure 3:**
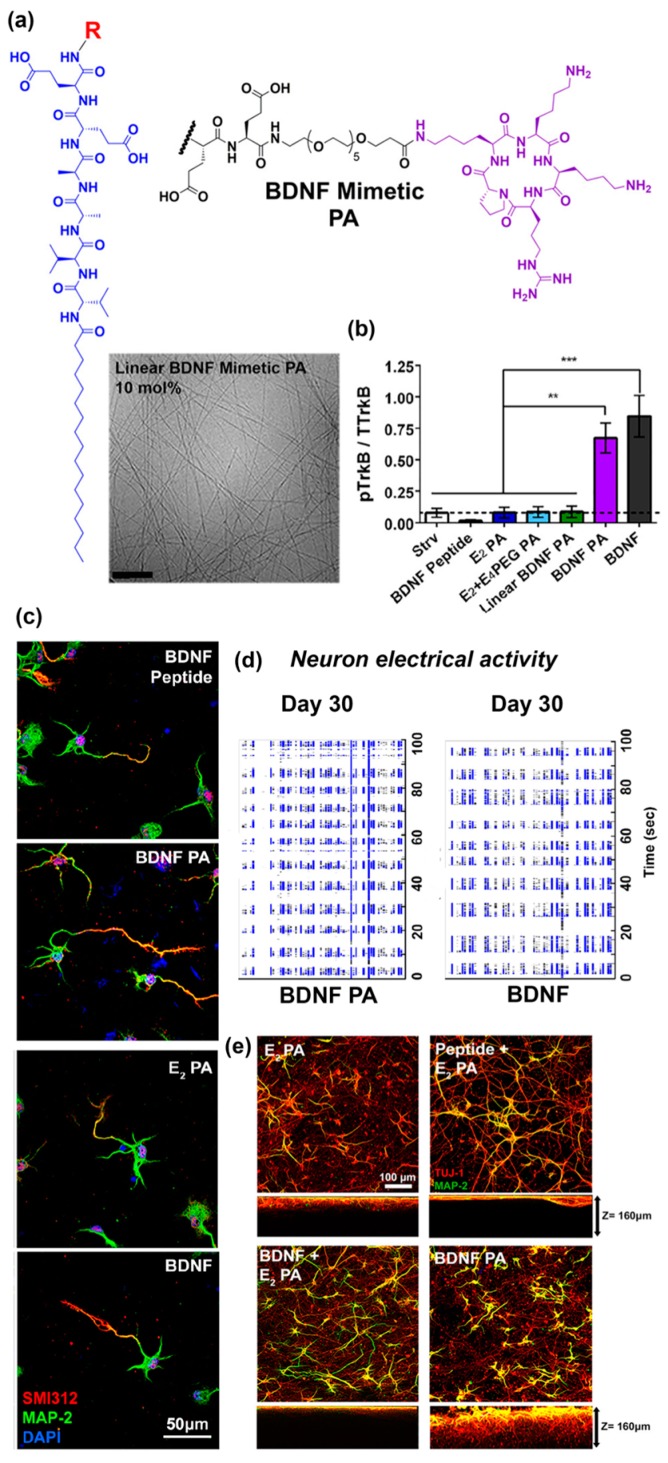
(**a**) Chemical structure of cyclic brain-derived neurotrophic factor (BDNF) mimetic peptide. Representative Cryo-TEM image of nanofibers derived from BDNF peptide amphiphiles (PA) co-assembled at 10 mol.% with E_2_ PA. β-sheet fibrous materials were obtained via assembly of BDNF PA and E_2_ PA. (**b**) Western blot densitometry analysis of phosphorylated tyrosine kinase B (p-TrkB) activation by cells treated with BDNF peptide, E_2_ PA, E_2_ + E_4_, linear BDNF, BDNF PA, and BDNF protein for 6 h in vitro. The BDNF PA-treated group showed comparable activation as cells treated with BDNF protein and significantly higher response relative to all other groups. (**c**) Representative confocal images of neuronal cells treated with BDNF peptide, E2 PA, BDNF PA, and BDNF protein for 24 h in vitro. Red fluorescence represents the axonal marker, pan-axonal neurofilament protein (SMI312), expressed on the cell surface, while green fluorescence represents the dendritic marker, microtubule associated protein 2 (MAP-2). The nucleus was stained using 4′,6-diamidino-2-phenylindole (DAPI) (blue fluorescence). A notable increase in axon length was observed when cells were treated with BDNF PA or BDNF protein, which suggests maturation of neuronal cells activated by TrkB receptor binding. (**d**) Raster plots show enhanced electrical activity for cells treated with BDNF PA or BDNF protein over 30 days of in vitro culture. (**e**) Representative confocal images of neuronal cells cultured on three-dimensional (3D) PA gel scaffolds for one week in vitro. Green fluorescence represents the dendritic marker MAP-2 on the cell surface, while red fluorescence represents the neuronal marker, neuron-specific class III beta-tubulin (Tuj-1). Extended neurites and a homogeneous neuronal network were observed in all gels tested. Furthermore, significantly higher MAP-2 expression was observed for cells cultured on BDNF PA gels or native BDNF + E_2_ PA gels when compared to other groups. (∗) *p* < 0.05, (∗∗) *p* < 0.01, and (∗∗∗) *p* < 0.001. Adapted with permission from [70]. Copyright 2018, American Chemical Society publishing.

Another recent example from Stupp and co-workers [70] reported self-assembled peptide amphiphiles that can activate the tyrosine kinase B (TrkB) receptor on primary cortical neurons by presenting a peptide that mimics brain-derived neurotrophic factor (BDNF) protein. The BDNF mimetic peptide was covalently conjugated to a peptide amphiphile (PA) consisting of two glutamic acid residues, two alanine residues, two valine residues, and an alkyl tail of 16 carbons (E_2_ PA) via a poly(ethylene glycol)_6_ (PEG_6_) spacer (Figure 3a). To form nanofibers, the BDNF mimetic PA was mixed with non-modified E_2_ PA at 10 mol.% (Figure 3a). BDNF-mimetic nanofibers activated the TrkB receptor of primary cortical neurons to a similar extent as BDNF protein in vitro. In contrast, nanofibers lacking the BDNF-mimetic peptide, as well as BDNF PA in the unassembled form, did not activate the TrkB receptor (Figure 3b). After 30 days of in vitro culture, cells treated with BDNF mimetic PA demonstrated enhanced expression of neuronal maturation markers compared to blank PA or linear BDNF PA (Figure 3c) and comparable electrical activity to cells treated with native BDNF protein (Figure 3d). Cortical neurons cultured on BDNF mimetic PA scaffolds demonstrated the highest degree of cell maturation among all of the scaffolds tested (Figure 3e). An interesting finding reported in this paper was that the BDNF mimetic peptide was only active when it was presented on a self-assembled nanofiber. The authors suggested that this may be due to multivalent presentation of the ligand on the nanofiber which facilitates receptor dimerization similar to native dimeric BDNF.

## 3. Moving Beyond Peptides as the Functional Ligand

The preceding section highlighted recent examples of using self-assembled peptide nanofibers as scaffolds to guide cell phenotype and function via presentation of peptide ligands that bind to specific cell surface receptors. This is a rich and active area of research that is well documented in other excellent recent reviews, which we direct interested readers to [29,71,72,73,74]. Here, we shift our focus toward the increasing use of self-assembling peptides to integrate proteins or carbohydrates into supramolecular biomaterials.

### 3.1. Glycosylated Nanomaterials Fabricated from Carbohydrate-Modified Self-Assembling Peptides

Highly abundant in nature, carbohydrates not only provide an energy source for cell metabolism, they also specifically interact with a broad range of biomolecules, including lectins, growth factors, and other carbohydrates [75,76]. Binding events involving carbohydrates are often weak, with dissociation constants in the milli- to micromolar range; however, carbohydrate-ligand interactions can be significantly enhanced by multivalency, often referred to as the “*glycocluster effect*” [77]. Inspired by this, conjugates of carbohydrates and peptides (i.e., “glycopeptides”) [78,79,80], as well as carbohydrates and polymers (i.e., “glycopolymers”) [81,82,83], began receiving attention as synthetic glycoclusters a few decades ago. With advances in chemistry, as well as increased understanding of lectin-glycan interactions, the focus shifted toward fabricating glycopeptides that selectively bind to specific lectins by tailoring carbohydrate chemistry and physical presentation [84,85]. For an overview of the synthetic methodologies used to prepare glycopeptides, we direct readers to an excellent review published elsewhere [86]. Here, we survey recent advances in glycosylated nanomaterials fabricated from self-assembling glycopeptides, which are finding increasing use as biomaterials that can regulate cell behavior through interactions with carbohydrate-binding proteins. 

One emerging application of glycopeptides is to create nanomaterials that can mediate cell adhesion or activate cell signaling events by interacting with carbohydrate-binding receptors [87,88,89]. For example, Guler and co-workers used glycopeptide amphiphiles to fabricate glycosaminoglycan (GAG)-like nanomaterials. The resulting assemblies bound to cluster of differentiation 44 (CD44) receptors and promoted chondrogenic differentiation of MSCs [88]. The same group also created an extracellular matrix (ECM)-mimicking scaffold using glycopeptide amphiphiles, which enhanced MSC adhesion. By altering the presentation of different functional groups, these scaffolds could induce differentiation of MSCs into brown adipocytes [89]. Additionally, they developed a self-assembled mannosylated peptide amphiphile decorated with antigen-mimetic GM3-lactone molecules as the basis for vaccines that target dendritic cells and induce their maturation [87].

**Figure 4:**
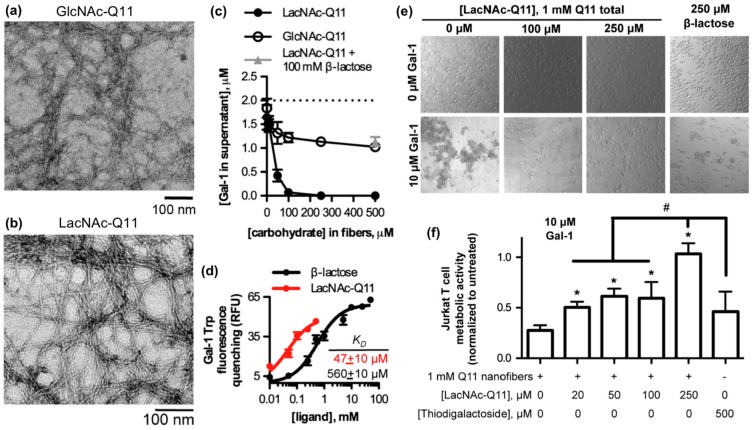
Glycopeptide nanofibers that bind and inhibit galectin-1. (**a**) TEM of *N*-acetylglucosamine (GlcNAc)-Q11 and (**b**) *N*-acetyllactosamine (LacNAc)-Q11 nanofibers assembled in aqueous buffer (pH 7.4). Images show similar fiber morphologies for GlcNAc-Q11 and LacNAc-Q11 fibers. (**c**) Binding of 2 μM Galectin-1 to LacNAc-Q11 (black circle) or GlcNAc-Q11 (hollow circle) nanofibers with 0–500 μM LacNAc or GlcNAc and (**d**) tryptophan fluorescence quenching of Galectin-1 by LacNAc-Q11 nanofibers or soluble β-lactose. Panel (**c**) also demonstrates inhibition of LacNAc-Q11 and Galectin-1 binding via β-lactose (triangle). (**e**) Bright-field photomicrographs of Jurkat T-cell agglutination and (**f**) metabolic activity of Jurkat T cells in culture media with or without Galectin-1 in the presence of Q11 nanofibers with different LacNAc content. LacNAc-Q11 nanofibers inhibited agglutination of Jurkat T cells induced by Galectin-1, whereas β-lactose was less effective. Likewise, LacNAc-Q11 nanofibers prevented the decrease in metabolic activity induced by Galectin-1, whereas thiodigalactoside was ineffective. * represents *p* < 0.05 compared to 0 μM LacNAc-Q11 or, # represents *p* < 0.05 compared to 250 μM LacNAc group, ANOVA with Tukey’s post-hoc. Adapted with permission from [94]. Copyright 2015, Springer US publishing.

Galectins are a family of soluble carbohydrate-binding proteins that regulate cell behavior in various healthy and pathological processes, such as integrin-mediated cell adhesion and migration [90], inflammation and its resolution [91], T-cell activation [92], and viral infection [93]. Galectins bind to β-galactosides, such as *N*-acetyllactosamine and related variants found on laminin, type IV collagen, and various cell membrane glycoproteins. Hudalla and co-workers used a peptide self-assembly strategy to develop synthetic glycoclusters that can bind to galectins and inhibit their activity as extracellular signals [94]. Their approach was based on a variant of the β-sheet fibrillizing peptide, QQKFQFQFEQQ (Q11), which has the monosaccharide *N*-acetylglucosamine (GlcNAc) conjugated to an asparagine residue added at the N-terminus of the peptide. GlcNAc-Q11 assembles into β-sheet nanofibers with similar morphology as Q11 nanofibers (Figure 4a). GlcNAc groups on the nanofiber can be converted to *N*-acetyllactosamine (LacNAc) via a glycosyltransferase enzyme in the presence of a sugar donor without disrupting nanofiber formation (Figure 4b). LacNAc-Q11 nanofibers bound Galectin-1 with higher affinity than GlcNAc-Q11 nanofibers or soluble β-lactose (Figure 4c,d). Due to this increased binding affinity, LacNAc-Q11 nanofibers inhibited T-cell agglutination and metabolic activity loss induced by Galectin-1 more effectively than soluble β-lactose or thiodigalactoside, a synthetic small-molecule Galectin-1 inhibitor (Figure 4e,f). 

One limitation of LacNAc is that it can interact with all members of the galectin family. To create nanofibers that selectively recognize Galectin-3, Hudalla and co-workers adapted their strategy to replace LacNAc on Q11 nanofibers with *N*,*N*′-diacetyllactosamine (LacDiNAc), a disaccharide that selectively binds to Galectin-3 [95]. LacDiNAc-Q11 nanofibers bound Galectin-3 with similar affinity as LacNAc-Q11 nanofibers but demonstrated no affinity for Galectin-1. LacDiNAc-Q11 nanofibers can inhibit T-cell apoptosis induced by Galectin-3; however, their results demonstrated that serum glycoproteins outcompete Galectin-3 binding to LacDiNAc-Q11 nanofibers, which diminishes their inhibitory activity. Competitive interactions between Galectin-3, serum glycoproteins, and synthetic multivalent glycoclusters may have important implications for developing better Galectin-3 inhibitors. 

**Figure 5:**
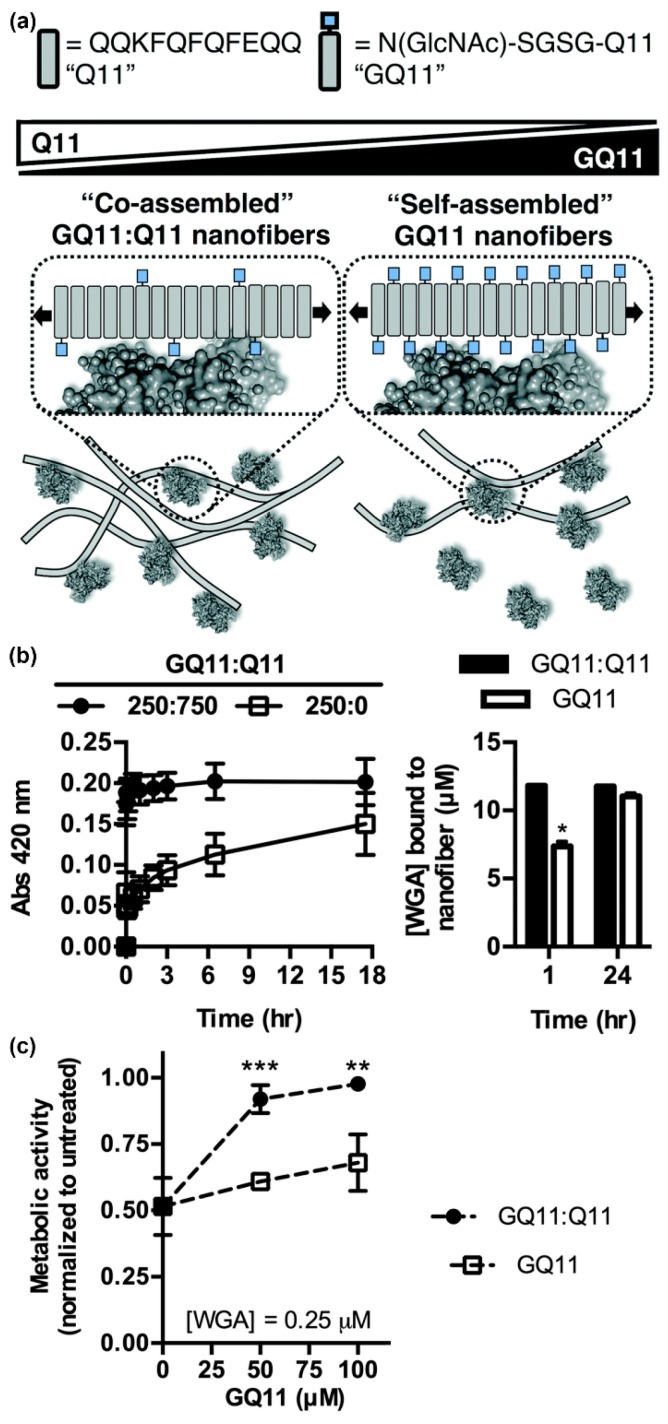
Influence of carbohydrate density and valency on lectin binding to glycopeptide nanofibers. (**a**) Schematic representation of self-assembled glycopeptide nanofibers with different carbohydrate densities and their potential interaction pattern with proteins. Bound proteins are expected to hide neighboring ligands on nanofibers with high carbohydrate density. (**b**) Turbidity (left) and co-precipitation assays (right) demonstrate that wheat germ agglutinin (WGA) binds faster to GlcNAc-Q11 and Q11 (1:3 ratio) mixed nanofibers (moderate density) compared to pure GlcNAc-Q11 nanofibers (high density). * represents *p* < 0.005, Student’s t-test. (**c**) Nanofibers with optimal carbohydrate density inhibited WGA-induced Jurkat T-cell death more effectively than nanofibers with high carbohydrate density. ** represents *p* < 0.01 and *** represents *p* < 0.001, Student’s *t*-test. Reproduced from reference [96] with permission from The Royal Society of Chemistry.

In addition to the carbohydrate type, the density and valency of carbohydrates in a glycocluster can also influence protein binding specificity and affinity. Using GlcNAc-Q11, Hudalla and co-workers studied relationships between lectin binding and carbohydrate display on peptide nanofibers (Figure 5a) [96]. Nanofibers with a range of carbohydrate densities and valencies were fabricated by mixing GlcNAc-Q11 and Q11 peptides at different molar ratios. Moderate carbohydrate densities provided optimal binding kinetics and extent of binding for both wheat germ agglutinin (WGA) and *Griffonia simplicifolia* II (GS II), independent of carbohydrate valency (Figure 5b). Due to the increased binding kinetics, nanofibers with moderate carbohydrate density inhibited T-cell apoptosis induced by WGA more effectively than nanofibers with high carbohydrate density at equivalent valency (Figure 5c). Collectively, these results demonstrated that interactions between self-assembled glycopeptide nanofibers and proteins are dependent on the avidity of carbohydrates rather than the absolute amount. Interestingly, this differs from results observed with glycopolymers and glyconanoparticles, where increased valency typically results in increasing affinity [97,98,99]. These findings highlight the benefit of supramolecular systems that allow for carbohydrate type, density, and valency to be easily and systematically varied to identify optimal protein binding characteristics.

**Figure 6:**
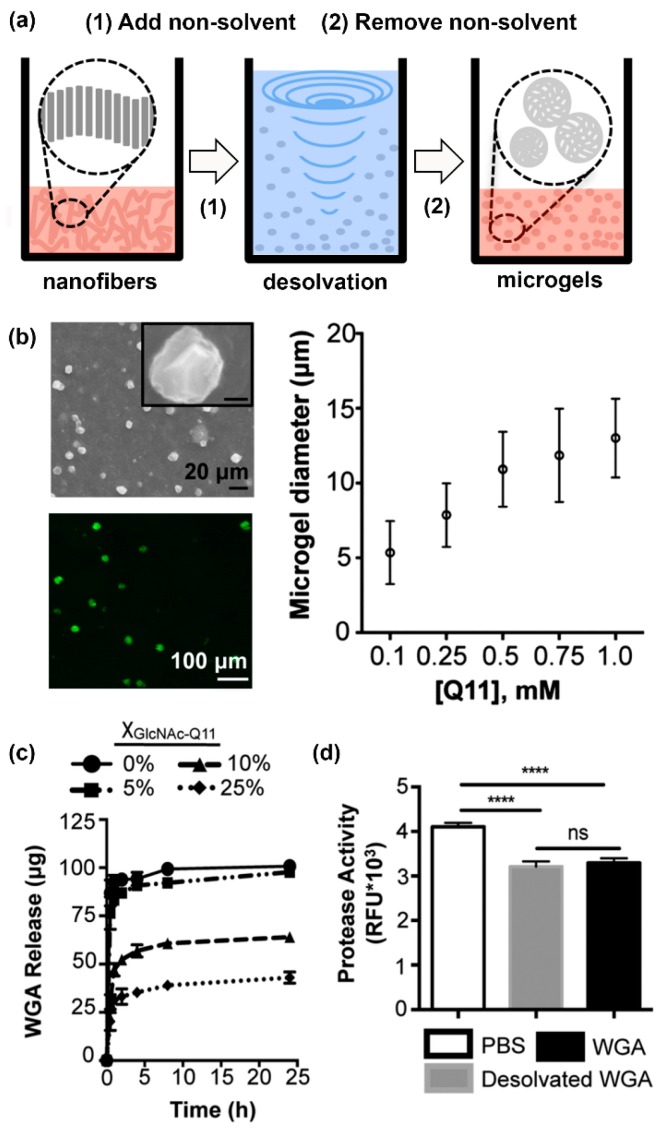
Lectin-releasing microgels fabricated from glycopeptide nanofibers. (**a**) Schematic representation of microgels fabricated from self-assembled peptide nanofibers via desolvation. (**b**) Microgels ranging from 5–12.5 µm in diameter can be fabricated by adjusting peptide concentration in solution. (**c**) WGA burst release curves from microgels with different GlcNAc content: 0% (circles), 5% (squares), 10% (triangles), or 25% (diamonds). Burst release decreased with increasing amount of GlcNAc-Q11. (**d**) Jurkat apoptosis induced by WGA released from Q11 microgels (gray), or stock WGA that was not subjected to desolvation (black), demonstrating that the released proteins were active. “ns” denotes *p* > 0.05 between indicated groups, **** indicates *p* < 0.001, ANOVA with Tukey’s post hoc. Adapted from reference [100] with permission from The Royal Society of Chemistry.

Finally, Hudalla and co-workers developed microgels for affinity-controlled release of lectins via desolvation of GlcNAc-Q11 glycopeptide nanofibers (Figure 6a) [100]. Microgels with different sizes can be prepared by adjusting peptide concentrations (Figure 6b). Microgels demonstrating tunable release of WGA can be prepared by varying the amount of GlcNAc-Q11 relative to Q11 (Figure 6c). WGA released from GlcNAc-Q11 microgels was biologically active, as demonstrated by its ability to induce Jurkat T-cell apoptosis in vitro (Figure 6d). These results demonstrate the potential to formulate glycopeptide nanofibers into controlled-release vehicles for the delivery of therapeutic lectin payloads.

**Figure 7:**
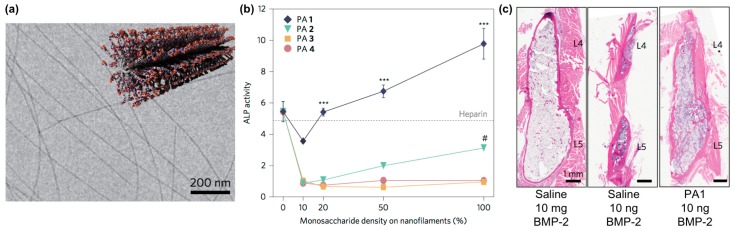
Sulfated glycopeptide amphiphile nanofilaments amplify BMP-2 activity. (**a**) Representative cryo-TEM image of self-assembled sulfated glycopeptide amphiphile nanofilaments formed in aqueous solution. The inset shows a cartoon representation of the self-assembled sulfated glycopeptide amphiphile nanofilament structure. (**b**) Alkaline phosphatase (ALP) activity in C2C12 cells treated with BMP-2 and glycopeptide amphiphile nanofilaments increased with increasing monosaccharide density; results from heparin control group (10 μg∙mL^−1^) are shown by the dashed line. It can be observed that the self-assembled trisulfated glycopeptide amphiphile nanofilaments (PA1) significantly increased the effect of BMP-2 on ALP activity. Two-way analysis of variance (ANOVA) with Bonferroni post hoc test, *** *p* < 0.001 compared with PAs 2–4, # *p* < 0.05 compared with PAs 3 and 4; (**c**) Representative hematoxylin and eosin (H&E) staining of sagittal cross-sectional images of bilateral fusion between L4 and L5 transverse processes. A comparable fusion was observed using PA1 and BMP-2 at 100-fold lower concentration. PA1: trisulfated 3,4,6S-*N*-acetyl glucosamine modified peptide amphiphile nanofilaments; PA2: monosulfated 6S-GlcNAc modified peptide amphiphile nanofilaments; PA3: monocarboxylated glucuronic acid-modified peptide amphiphile nanofilaments; PA4: uncharged GlcNAc modified peptide amphiphile nanofilaments. Adapted with permission from [101]. Copyright 2017, Nature publishing.

Self-assembling glycopeptide amphiphiles are also finding use as building blocks for supramolecular nanomaterials that can amplify the activity of carbohydrate-binding proteins. For example, Stupp and co-workers designed nanofilaments that present sulfated carbohydrates as biomaterials that can bind to bone morphogenetic protein 2 (BMP-2) to amplify its activity during bone regeneration (Figure 7a) [101]. Specifically, they used the copper(I)-catalyzed alkyne-azide cycloaddition (CuAAC) click reaction to conjugate different monosaccharides onto peptide amphiphiles [102]. The multivalency afforded by these glycosylated nanofilaments was intended to mimic natural highly sulfated complex polysaccharides that bind to various growth factors. These nanofilaments amplified the activity of BMP-2 in a monosaccharide density-dependent manner, as measured through up-regulation of alkaline phosphatase activity (Figure 7b). In vivo studies demonstrated that trisulfated self-assembled glycopeptide nanofilaments could decrease the effective BMP-2 dose required for bone fusion by 100-fold compared to using BMP-2 alone (Figure 7c).

### 3.2. Incorporating Folded Proteins into Supramolecular Nanomaterials 

The extracellular matrix (ECM) is a supramolecular assembly consisting of various proteins, glycoproteins, glycosaminoglycans, and proteoglycans. Furthermore, individual ECM components, such as fibrillar collagens and elastin, are assemblies of individual protein subunits [103,104]. Inspired by these natural processes, strategies to integrate proteins into synthetic supramolecular biomaterials are receiving increasing attention [105,106]. Silk proteins and related mimics are now used as biomaterials for diverse purposes in various biomedical fields [107,108]. Similarly, supramolecular biomaterials based on elastin-like polypeptides are commonly employed as drug delivery vehicles [109]. Self-assembled collagen mimics are also gaining interest as nanomaterials [65]. In this section, we highlight recent advances in fusing self-assembling peptides to proteins to create nanomaterials with advanced functional properties.

**Figure 8:**
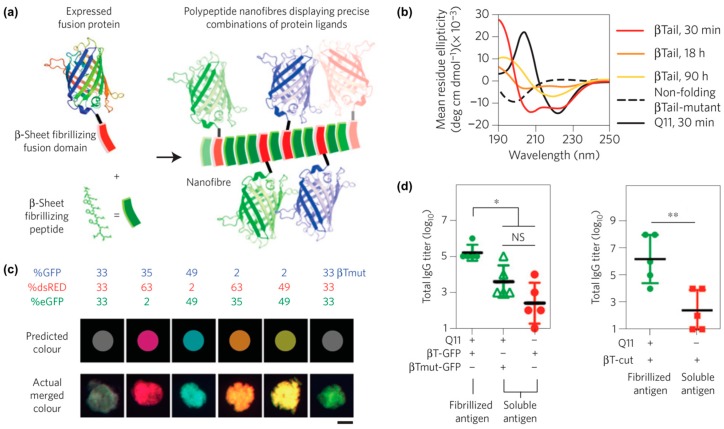
Assembly of peptides and proteins into multi-functional nanofibers. (**a**) Graphical representation of fusion proteins co-assembled with Q11 into β-sheet nanofibers. (**b**) Circular dichroism curves of the secondary structure of the β-Tail peptide at different time points showing the transition from an α-helix to a β-sheet. A mutated β-Tail, used here as a negative control, adopted a random coil structure. (**c**) Fluorescence images of red, green, and blue β-Tail proteins co-assembled into Q11 microgels at a predetermined ratio. Top row shows the predicted merged color, while the bottom row shows the actual merged color. A mutated β-Tail led to mismatch between predicted and experimental colors, demonstrating that integration of the β-Tail proteins is via the assembly process rather than physical absorption. (**d**) Antibody responses in C57BL/6 mice treated with Q11 nanofibers bearing β-Tail-GFP (left) or β-Tail-cutinase (right). Significantly higher antibody titers were observed when animals received protein assembled into Q11 nanofibers. * *p* < 0.05, ** *p* < 0.01, NS, no significant differences (*p* > 0.05). ANOVA with Tukey’s post-hoc (left) and Student’s t-test (right). Adapted with permission from [110]. Copyright 2014, Nature publishing.

One example from Collier and co-workers reported self-assembled peptide-based nanomaterials with multiple different co-integrated proteins (Figure 8a) [110]. Each protein was fused to a “β-Tail” (MALKVELEKLKSELVVLHSELHKLKSEL) tag, which is a peptide that undergoes slow transition from an α-helix to β-strands that form nanofibers (Figure 8b). β-Tail fusion proteins assemble with the β-sheet fibrillizing peptide Q11 to form nanofibers modified with active protein domains. For example, fluorescent nanofibers can be prepared by assembling β-Tail-GFP with Q11, while nanofibers with hydrolase activity can be fabricated by assembling β-Tail-cutinase with Q11. Various β-Tail fusion proteins can be co-assembled to fabricate multifunctional nanofibers. The relative abundance of each protein can be independently varied to fabricate nanofibers with tunable properties, as demonstrated by materials with a range of fluorescent hues that correspond to the feed ratio of red, green, and blue fluorescent proteins co-assembled with Q11 (Figure 8c). Lastly, in vivo studies showed that antibodies can be raised against β-Tail fusion proteins assembled into Q11 nanofibers (Figure 8d), which demonstrates the potential of this platform for developing a multi-antigen vaccine.

**Figure 9:**
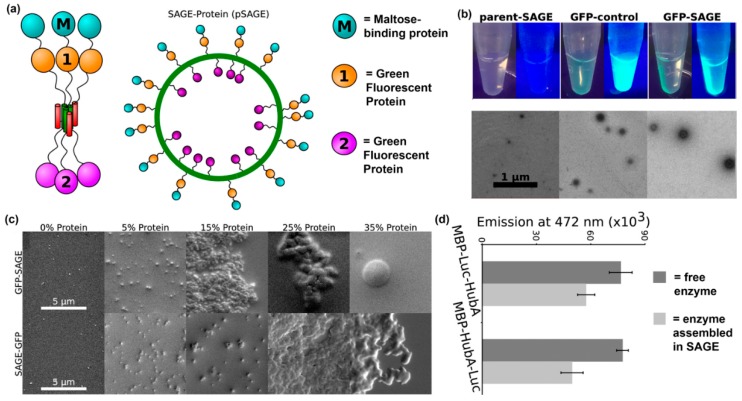
Self-assembled cages displaying protein domains. (**a**) Schematic representation of a cage formed from self-assembling peptide–protein conjugates. Proteins can be displayed within the core or on the surface of the cage using this methodology. (**b**) Representative fluorescence still images and transmission electron microscopy images of assembled peptide–protein cages. Particles assembled in the presence of GFP fusion proteins were fluorescent. Particles with average diameters of ~100 nm and uniform morphologies were observed. (**c**) The average particle diameter depended on protein orientation. Slightly larger particles were obtained when proteins were conjugated on the outside of the particles. (**d**) Representative SEM images of assembled particles incorporating 0%, 5%, 15%, 25%, and 35% (volume ratio) peptide–protein conjugates. Individual particles were observed below 15% protein loading, while aggregation occurred at higher concentrations. (**e**) Bioluminescence emission at 472 nm from 5% *Renilla* luciferase assembled into cages (light gray) or free in solution (dark gray). Differences between assembled and soluble enzyme were insignificant. Adapted with permission from [111]. Copyright 2017, American Chemical Society publishing.

Another recent example of installing proteins into supramolecular nanomaterials via fusion to a self-assembling peptide was reported by Woolfson and colleagues. In this approach, they fabricated nanoreactors by displaying proteins on cages assembled from peptides that form α-helical coiled-coils [111,112] (Figure 9a,b). The orientation of a protein on a self-assembled cage can be controlled by conjugating it to either the N- or C-terminus of the coiled-coil peptide, as demonstrated by differences in average cage diameter. The amount of protein loaded into self-assembled cages can also be varied by changing the ratio of protein-modified and unmodified peptide, with a maximum of 15% loading before significant cage aggregation was observed (Figure 9c). Finally, activity of the loaded proteins was characterized by measuring bioluminescence emission from *Renilla* luciferase displayed on self-assembled cages in the presence of coelenterazine. Their results showed that luminescence emission by enzyme localized to the core or surface of cages was comparable to free enzyme (Figure 9d). 

**Figure 10:**
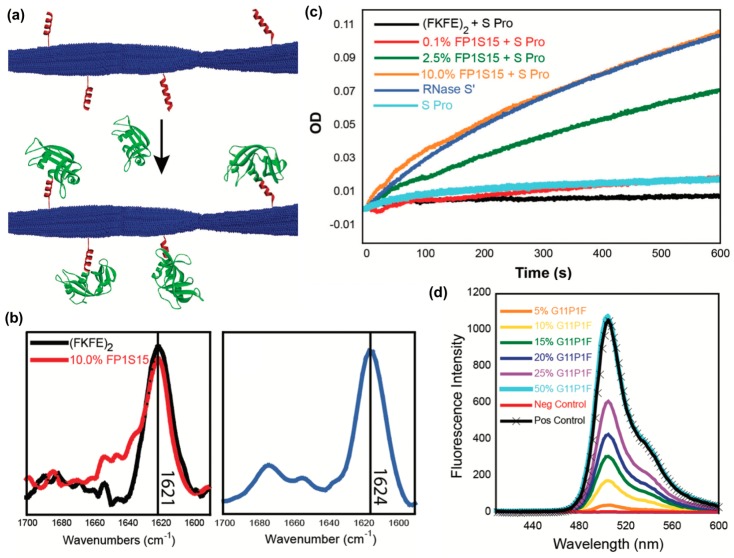
Immobilizing proteins onto peptide nanofibrils via split tag. (**a**) Graphical representation of non-covalent protein conjugation onto self-assembled peptide nanofibrils using a split protein strategy. Blue: a self-assembling peptide (Ac-(FKFE)_2_-NH_2_); red: peptide tag; green: split protein fragments. (**b**) Fourier-transform infrared (FT-IR) spectra indicating that 10% co-assembled FP1S15/Ac-(FKFE)_2_-NH_2_ fibrils and 20% G11P1F/Ac-(FKFE)_2_-NH_2_ fibrils (20% G11P1F) adopt a β-sheet secondary structure. (**c**) Optical density curves of cytidine 2′,3′-cyclic monophosphate hydrolysis catalyzed by self-assembled nanofibrils with different amounts of ribonuclease S tag in the presence of ribonuclease S′. (**d**) Spectra of green fluorescence produced by self-assembled nanofibrils with different amounts of GFP 11 tag in the presence of GFP 1-10. Control spectra are GFP 1–10 alone (red) and GFP 1–10 + GFP 11 (black). Reproduced from reference [113] with permission from The Royal Society of Chemistry.

A third recent example reported the fabrication of peptide nanofibrils displaying functional proteins using a split-tag method [113]. In particular, two different peptide tag sequences that recognize either RNase S-protein or a GFP fragment (S-peptide and GFP 11, respectively) were conjugated to the β-sheet fibrillizing peptide (FKFE)_2_ via a PEG linker (Figure 10a). Both (FKFE)_2_ variants self-assembled into β-sheet rich fibrillar structures (Figure 10b). Nanofibrils bearing S-peptide catalyzed the hydrolysis of cytidine 2′,3′-cyclic monophosphate in the presence of the split protein RNase S′, and the rate of reaction increased with increasing tagging ratio (Figure 10c). Similarly, nanofibrils bearing GFP 11 were fluorescent in the presence of split GFP, and the amount of fluorescence increased with the increasing amount of GFP 11 on the fibrils (Figure 10d). 

**Figure 11:**
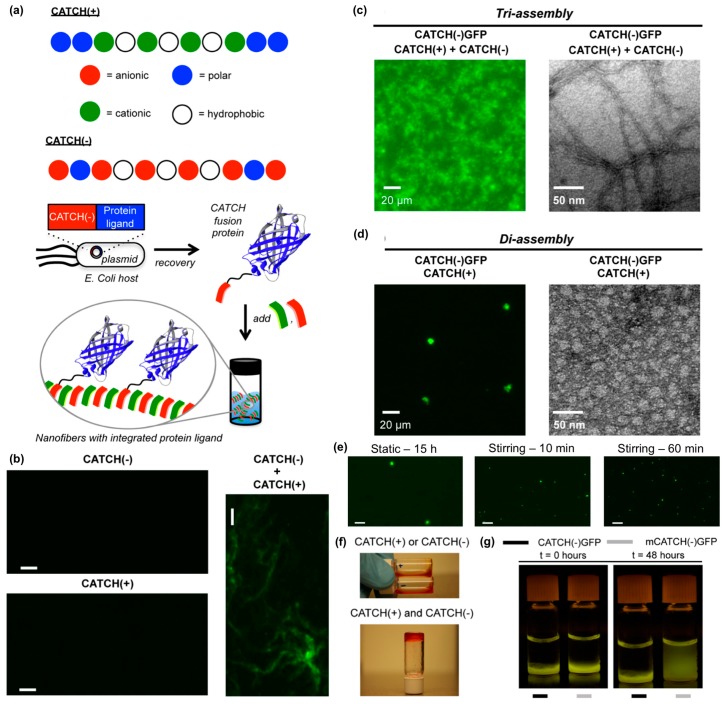
Co-assembling peptide tags for protein immobilization within nanofibrillar hydrogels. (**a**) Schematic representation of the Co-Assembly Tags based on CHarge complementarity (CATCH) nanofiber system. (**b**) Fluorescence photomicrographs of solutions containing Thioflavin T (ThT) and CATCH(−) only, CATCH(+) only, or an equimolar mixture of CATCH peptides. Nanofibrous structure was only observed in mixtures containing both peptides. Fluorescence photomicrograph (left) and transmission electron micrograph (right) of (**c**) CATCH(−), CATCH(+), and CATCH(−)GFP tri-assemblies, or (**d**) CATCH(+) and CATCH(−)GFP di-assemblies. Triassemblies formed nanofibrous structures, while diassemblies formed micron-sized particles. (**e**) Fluorescence photomicrographs of di-assembly microparticles after 15 h of static incubation (left), 10 mins of stirring (middle), and 60 mins of stirring (right). A notably higher number of microparticles having a smaller diameter formed with stirring compared to static control group. (**f**) Digital still images of aqueous buffered solutions of CATCH(+) and CATCH(−) alone (top), and hydrogel formed after mixing at a 1:1 (*v*/*v*) ratio (bottom). * Red food coloring was added to samples for ease of viewing. (**g**) Blue-light trans-illuminated digital still images of hydrogels fabricated by mixing CATCH(−) and CATCH(+) with 0.75 µM CATCH(−)GFP or 0.75 µM mutant CATCH(−)GFP (“mCATCH(–)GFP”) before and after 48 hours of incubation in excess 1× phosphate-buffered saline (PBS). CATCH(–)GFP was retained within the hydrogel over time while mCATCH(–)GFP was released into the surrounding medium. Scale bar = 20 μm in (**b**) and (**e**). Adapted with permission from [114]. Copyright 2016, Springer US publishing.

Hudalla and co-workers reported a co-assembly strategy to fabricate β-sheet nanofibers with pendant protein domains [114]. Co-assembly tags based on charge complementarity or “CATCH peptides”, are anionic (“CATCH(−)”) and cationic (“CATCH(+)”) variants of the β-sheet fibrillizing peptide Q11 (Figure 11a). CATCH peptides resist self-assembly due to strong electrostatic repulsion, yet co-assemble into two-component nanofibers when mixed due to charge complementarity (Figure 11b). This allows for CATCH fusion proteins to be expressed from recombinant DNA by bacteria without premature assembly or aggregation (Figure 11a). In turn, CATCH fusion proteins added to mixtures of CATCH peptides incorporate into the resulting nanofibers (Figure 11a). For example, CATCH(+), CATCH(−), and a CATCH(−)GFP fusion protein co-assemble to form fluorescent nanofibers (Figure 11c), while binary mixtures of CATCH(−)GFP and CATCH(+) peptide co-assemble into micron-sized fluorescent particles (Figure 11d). The transition from particle to nanofiber morphology depends on the feed ratio of CATCH(−) and CATCH(+) peptides mixed with CATCH(−)GFP, and the size of the microparticles formed can be varied by stirring binary mixtures of CATCH peptides and fusion proteins (Figure 11e). At higher concentrations, CATCH(+) and CATCH(−) form hydrogels (Figure 11f). Ternary mixtures of CATCH(+), CATCH(−), and CATCH(−)GFP yield fluorescent hydrogels that retain GFP over many days, while hydrogels assembled from CATCH(+), CATCH(−), and a GFP with a mutated CATCH tag release GFP into surrounding aqueous media over time (Figure 11g). Together, these observations demonstrate the potential of CATCH peptides as fusion tags to immobilize functional proteins within nanofibrillar hydrogel scaffolds. 

**Figure 12:**
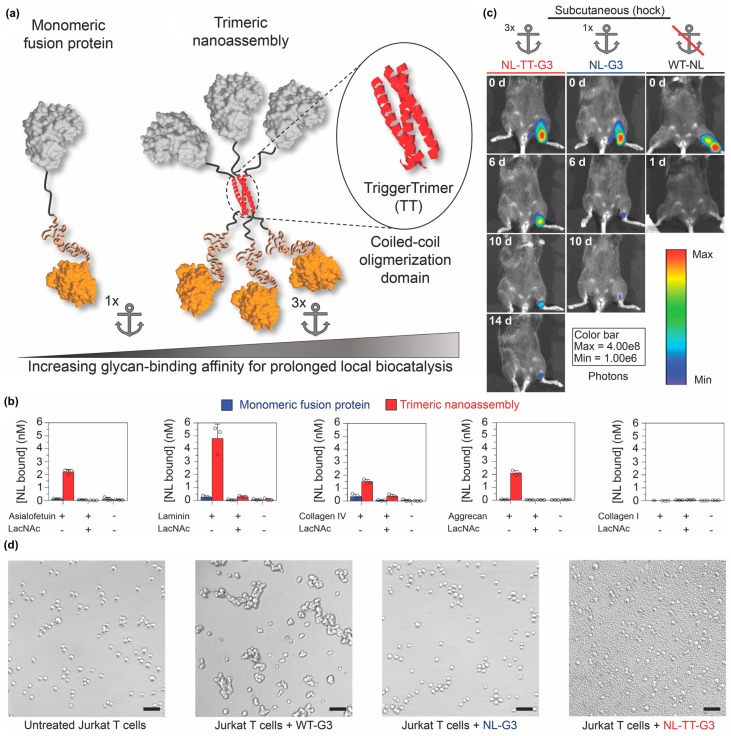
Locally anchoring enzyme nanoassemblies to tissues via Galectin-3. (**a**) Schematic representation of protein nanoassemblies fabricated using peptides that form an α-helical coiled-coil. (Left) A monomeric fusion protein consisting of an enzyme linked to the N-terminal domain of Galectin-3 (G3) via a flexible peptide linker. (Right) Trimeric nanoassembly formed by inserting the TriggerTrimer α-helical coiled-coil domain between the enzyme and G3 domains. The trimeric nanoassembly has higher glycan-binding affinity than the monomeric fusion protein due to multivalent avidity effects. (**b**) Carbohydrate-binding properties of monomeric G3 fusion proteins and trimeric nanoassemblies. (**c**) Bioluminescence images at various time points of mice that received trimeric nanoassemblies of NanoLuc and Galectin-3 (NL-TT-G3), a monomeric fusion protein of NanoLuc and Galectin-3 (NL-G3), or wild-type NanoLuc (WT-NL) (equivalent moles of NL) injected subcutaneously into the hock. Representative images show prolonged residence of trimeric nanoassemblies at the injection site compared with other groups. (**d**) Bright-field micrographs of Jurkat T cells incubated with PBS (untreated, negative control), wild-type Galectin-3 (WT-G3) (positive control), NL-G3, or NL-TT-G3 for 4 h, which demonstrated that monomeric fusion proteins and trimeric nanoassemblies lacked the activity for inducing T-cell apoptosis that is characteristic of WT-G3. Adapted with permission from reference [115] under Creative Commons Attribution 4.0 International license. Copyright 2018. Nature publishing.

Self-assembling peptides can also be used to organize proteins into other nano-scale architectures in addition to nanofibers and nanovesicles. For example, Hudalla and colleagues created nanoassemblies by fusing an enzyme to Galectin-3 via a peptide that forms an α-helical coiled-coil [115] (Figure 12a). Galectin-3 is a protein that binds carbohydrates found on the cell surface and within the extracellular matrix of mammalian tissues, including *N*-acetyllactosamine and related variants, as well as chondroitin and heparan sulfate glycosaminoglycans [116,117]. Trimeric nanoassemblies having three enzymes and three Galectin-3 domains bound carbohydrates with significantly higher affinity than a monomeric fusion of enzyme and Galectin-3 connected by a flexible linker (Figure 12b). When injected at different tissue sites, trimeric nanoassemblies of NanoLuc^TM^ luciferase (Promega Corporation, Madison, WI, USA) and Galectin-3 persisted for two weeks, whereas the monomeric fusion was retained for approximately one week. In contrast, native NanoLuc^TM^ cleared within one day (Figure 12c). Importantly, unlike wild-type Galectin-3, which forms higher-ordered oligomers that can induce T cell death, trimeric nanoassemblies did not induce T-cell apoptosis (Figure 12d). Collectively, this report demonstrates that the carbohydrate-binding properties of Galectin-3 can be harnessed to anchor enzymes at tissue injection sites independently of Galectin-3 signaling activity. 

## 4. Future Directions

Self-assembling peptides have significantly advanced the state of the art of nano-scale biomaterials over the last few decades, as exemplified by their increasing use in diverse applications such as drug delivery, tissue engineering, regenerative medicine, vaccines, and stimuli-responsive biomaterials [16,20,73]. Essential to this breadth of use is the ability to easily install different functional ligands into supramolecular biomaterials by simply conjugating them to a self-assembling peptide. Building upon early iterations, in which the functional ligands were often peptides or small molecules, the examples surveyed herein highlight the growing use of self-assembling peptides as handles to incorporate folded proteins or carbohydrates into supramolecular architectures. The sophisticated biochemical properties of proteins and carbohydrates open up exciting opportunities to develop novel biomaterials with unprecedented functional capabilities. We envision that continued progress using self-assembling peptides to integrate diverse types and combinations of biologically active ligands into supramolecular biomaterials will greatly advance their use in existing and emerging areas of biomedicine and biotechnology.
